# Global Pathways to Thriving Children: Implementing Large-Scale Multicomponent Nutrition and Child Development Programs^☆^

**DOI:** 10.1016/j.tjnut.2026.101677

**Published:** 2026-06-19

**Authors:** Maureen M Black, Chessa K Lutter

**Affiliations:** 1 Department of Pediatrics, University of Maryland School of Medicine, Baltimore, MD, United States; 2 Independent Consultant, Harbert, MI, 49115, United States

**Keywords:** thriving children, nurturing care, implementation science, multicomponent nutrition and development programs, low- and middle-income countries, sustainability

## Abstract

Thriving children are likely to develop the human capital necessary to become healthy, competent, and economically stable adults, beginning with achieving expected rates of growth and development as infants. However, millions of young children in low-and middle-income countries are not thriving, largely due to nutritional deficiencies and lack of learning opportunities, often caused by poverty. Although nutrition interventions can support children’s growth and nutrition and child development and parenting interventions can support children’s learning and responsive caregiving, there are few large-scale, multicomponent programs that can support both nutrition and child development. Informed by concepts of nurturing care and principles of implementation science, this article provides rational and systems-based criteria for implementing large-scale, multicomponent nutrition and child development programs for children in disadvantaged environmental conditions. The criteria include the following—*1*) goals: set goals informed by theory and evidence-based interventions; *2*) equity: reach vulnerable children and implement developmentally and culturally appropriate programs; *3*) quality: establish workforce development, support resources for delivery, and monitor for accountability; *4*) engagement: engage participants, communities, and stakeholders in active roles; *5*) outcome: implement rigorous and easy-to-use outcome assessments; and *6*) sustainability, strive for sustained funding and update policies, practices, and laws. The system is coordinated by a multicomponent partner management team that operates in a continuous data-driven feedback loop, including government representatives and community participants. Building on the strengths of expertise in single sector nutrition and child development intervention research, the conclusion includes a call to action for the implementation of multicomponent large-scale programs for young children. An increase in children thriving during early childhood increases their likelihood of achieving health and developmental potential that can lead to human capital with extensions to economic productivity, positive quality of life, and sustainability for children, families, and countries.

## Introduction

### Thriving children

Thriving children have excellent health, grow as expected, meet developmental expectations, and bring joy to their families. Families’ desire to raise thriving children is universal, illustrated by examples of thriving children among families who vary in race, ethnicity, cultural traditions, religion, and many other conditions [1]. However, millions of young children throughout the world are experiencing deficiencies in nutrition and/or child development that preclude thriving [2].

Among children under age 5 y in low- and middle-income countries (LMICs), in 2022, 23% experienced stunting [3] and 40% had anemia, often caused by iron deficiency [3]. Using stunting and poverty as proxy measures, 43% were at risk of not meeting their developmental potential [2], and using the Early Child Development Index, 25% experienced suspected developmental delays [4]. Although data are often aggregated for children under age 5 y, for millions of children, the deficiencies begin prior to birth, with over one-quarter of live births in 2020 born preterm (8.8%), small-for-gestational-age (16.3%), or both (1.1%), increasing their vulnerability [5]. Early nutritional and developmental deficiencies represent a global crisis by jeopardizing children’s ability to thrive and endangering their ability to achieve the quality of life, economic stability, competence, and commitment as adults, necessary to achieve and maintain the Sustainable Development Goals.

To address deficiencies in nutrition and child development, effective interventions implemented globally have reduced stunting [6] and anemia [7] and improved children’s cognitive, motor, and socioemotional development [[8], [9], [10]]. In addition, multicomponent nutrition interventions [11] and multicomponent child development interventions [12] have been effective. Scaling successful interventions into large-scale programs requires planning, collaboration, and management [13], as shown in country-wide nutrition programs [14] and child development programs [15].

The oldest and primary example of a national multicomponent nutrition and child development program is the Integrated Child Development Services (ICDS) scheme, initiated by the government of India in 1972. The goal of the ICDS is to improve the nutrition, health, and development of children from birth to age 6 y and to support pregnant and breastfeeding women [16]. As a state-run program, coordinated by the Ministry of Women and Children, the ICDS operates through small Anganwadi Centers located in villages throughout the country that provide supplementary nutrition, preschool nonformal education, nutrition and health education, immunizations, health checkups, and referrals. In 2013, the National Food Security Act made ICDS-provided meals a universal entitlement, strengthening the nutrition component of the ICDS. Without legislative support, the child learning and education components are less strong than the nutrition components [16]. Based on India’s National Family Health Survey, ICDS participation among children aged 6 to 59 mo increased from 58% in 2015 to 2016 to 71% in 2019 to 2021, with a corresponding decrease in the prevalence of underweight, from 37% to 32% over the same period [17]. State management and investments in ICDS vary across states, with corresponding differences in the quality and impact of the program [16].

There are few other multicomponent programs that address both nutrition and child development, and none with the history, government support, and reach of the ICDS. Our article provides rational and systems-based criteria for implementing large-scale, multicomponent nutrition and child development programs to promote thriving among children in disadvantaged economic and environmental conditions. We begin with the rationale for focusing on postnatal thriving from birth to 24 mo-of-age (prenatal interventions are beyond the scope of the current study) and then present criteria needed for the successful and sustainable implementation of large-scale programs that improve young children’s nutrition and development [18]. We close with a discussion that addresses potential partners for large-scale multicomponent programs, potential threats, and conclude with a call to action.

### Rationale for focusing on thriving during early childhood

Thriving is dependent on healthy neural development, which begins prenatally with extension through infancy and childhood. Critical aspects of brain development are activated by time-dependent organizational processes that are highly sensitive to nutrients and to the caregiving environment [19]. Deficiencies in specific nutrients and aspects of infant and young child caregiving can result in long-term negative consequences to the developing neural system that can undermine functional outcomes, including academic performance, psychosocial development, and economic productivity [20,21]. The heightened neural plasticity during early childhood provides an opportunity for effective nutrient and caregiving programs to prevent or reduce negative sequelae associated with deficiencies and to advance children’s emerging language and cognitive skills. Neural plasticity decreases over time, such that programs during infancy may have greater impact on the neural system than programs later in childhood [22]. Although programs beyond infancy may improve children’s functioning, their efficacy is diminished at preventing or reducing the impact of early deficiencies on the neural system. Thriving through infancy positions children to develop the physical, neurological, cognitive, and psychosocial skills that are foundational for subsequent health, quality of life, and economic productivity [23].

## Systems-Based Criteria for Successful Implementation and Sustainability of Large-Scale Multicomponent Programs to Improve Young Child Nutrition and Development

**FIGURE 1:**
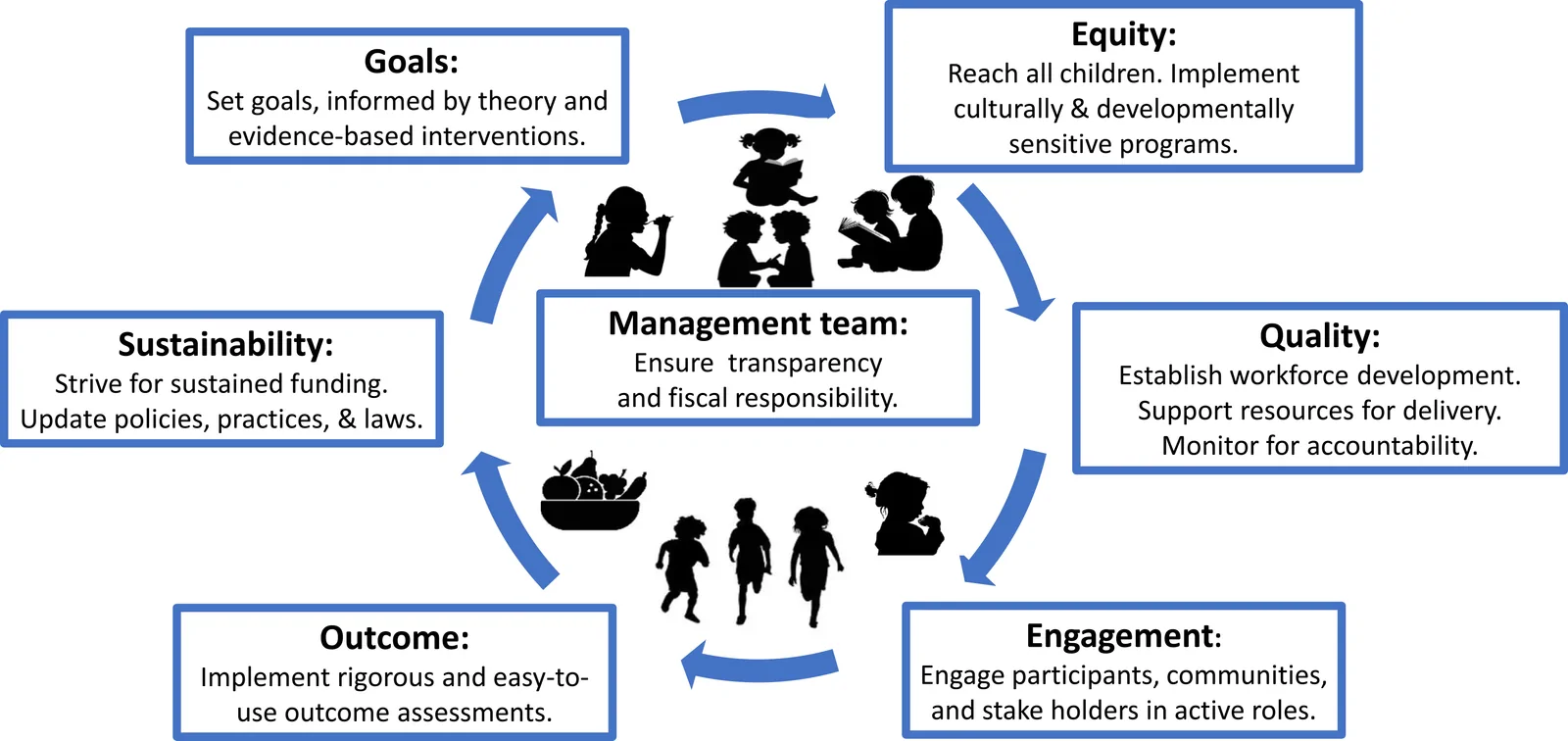
Systems-based criteria for implementation and sustainability of large-scale multicomponent programs to improve young children’s nutrition and development.

Figure 1 presents the systems-based criteria for successful promotion of thriving among children in disadvantaged environmental conditions by improving their nutrition and development. As with all systems, the criteria are interrelated, all are necessary, and if one falters, the entire system is jeopardized. The system is coordinated by a multicomponent partner management team and organized through 6 criteria, informed by nurturing care concepts and principles of implementation science [2,18].

Applied to thriving, implementation science is a system of methods to integrate evidence-based nutrition and child development practices into ongoing healthcare or social protection systems, with the overall impact of increasing children’s nutrition and child development [18]. Implementation science methods support program impact by addressing multiple criteria to ensure that programs are equitable and reaching children in greatest need, including high-quality implementation, workforce development, accountability, satisfaction, and sustainability. Implementation science builds participant and community trust by incorporating participants as partners in multiple phases of implementation and encouraging community mobilization such that activities to promote children’s thriving often originate in communities and bring excitement and enthusiasm to communities [23]. By committing to a responsive learning system, programs prepare for sustainability by managing decisions through data, building communication and feedback mechanisms and aligning with practices, policies, and laws with accountability.

### Criterion 1: goals

Goals drive the organization of programs and are typically informed by theory and evidence-based interventions. The nurturing care framework (NCF) is the underlying theory for the developmental progression of thriving [24]. As a system, nurturing care is characterized by a stable home environment that is adequately resourced and provides ongoing opportunities for 5 critical components for children, protection, health, nutrition, learning opportunities, and responsive caregiving [25]. All components are necessary and interrelated, laying the groundwork for a multicomponent program that advances children’s nutrition and development in a setting that provides safety, stability, and access to health care. The NCF was originally developed for children aged <3 y, but the principles extend through adolescence [26].

The WHO, UNICEF, and World Bank Group have embraced the NCF and encouraged countries to implement the NCF’s structure to inform and plan services to support children and families [24]. Although the implementation and monitoring of the NCF are organized and managed by countries, often guided by principles of implementation science, findings have shown uneven emphasis on nutrition and child development. For example, a review of policy documents in Kenya related to the components of nurturing care found implementation patterns that supported health and nutrition, with limited regard for early learning, responsive caregiving, and safety and security [27]. In another example, using data from the multiple indicator cluster surveys, 10 indicators of nurturing care related to learning opportunities (e.g., participation in early childhood care and education; access to adequate water, sanitation, and hygiene; and absence of physical punishment) were gathered from families of preschool-aged children from 31 LMICs [28]. Children’s development, measured with the early childhood development index, was positively associated with learning opportunities. Although these programs addressed children’s nutrition and learning, respectively, multicomponent programs that include both nutrition and child development are needed to promote thriving.

### Criterion 2: equity

Equity is a critical aspect of the NCF, with the objective of reaching all children, including the most vulnerable, and implementing programs that are culturally and developmentally sensitive [29]. There is limited information on whether NCF policies and programs are reaching hard to reach children or meeting the needs of children with disabilities and other special needs [30]. Children in rural and impoverished settings, children in conflict areas, and children with disabilities and other special needs may be excluded from programs, either inadvertently because they are less likely to access such programs [31] or purposively because they require specialized services. Understanding the reach of programs is a critical aspect of implementation science and often a priority of donors, governing organizations, and communities. Equity also includes addressing programs’ sensitivity to communities’ cultural variability and children’s changing developmental capabilities.

#### Cultural variability

Families have principal roles in young children’s daily care and are therefore primary participants of most nutrition and child development programs. Cultural beliefs and barriers about caring for children can result in large variability in foods that are offered to children and how practices related to feeding and caregiving are implemented. Practices that are acceptable and encouraged in some communities or families, such as promoting young children’s autonomy and self-regulation, may be perceived as inappropriate in others [32]. Programs that have been imported from other sites without local adaptation and consultation may not be consistent with local cultural traditions, potentially undermining program acceptance and impact [33].

In addition to caring for their children, caregivers’ commitments often include multiple household responsibilities and increasingly include external employment [34], thus posing significant challenges to caregiving [35]. Understanding the range of caregivers’ daily activities and their beliefs and priorities about feeding and playing with their children can be instrumental in developing programs that are consistent with caregivers’ cultural beliefs and time constraints and, therefore, likely to be successfully implemented. Formative qualitative methods, including observations, focus groups, and interviews to understand cultural practices, as well as involving families and communities in planning strategies, can avoid or overcome potential cultural barriers.

#### Developmentally sensitive

Children’s development through the first few years progresses from requiring total care in all areas to multiple areas of independence, including consuming the family diet, running, talking, and solving simple problems. As such, effective programs to promote children’s nutrition and development incorporate the expected developmental changes that occur as children age. Effective learning strategies for children include active engagement in activities and communication, with opportunities to observe and model from others, to use their curiosity to explore, and to establish mastery as they acquire new skills [36]. The learning process occurs through multiple daily activities, including playing and feeding.

Responsive caregiving, an essential component of the NCF, refers to caregivers noticing and responding to children’s verbal and nonverbal cues in a developmentally appropriate and nurturance manner [37]. Sometimes described as “serve and return,” mutually responsive caregiver–child interactions facilitate children’s attachment, cognitive development, and self-regulation [38]. Responsive feeding, a derivative of responsive care, follows a similar pattern [39]. When caregivers recognize children’s signals of hunger and satiety and respond appropriately, children learn to eat in response to physiological and developmental needs, which promotes self-regulation [40]. In contrast, feeding interactions dominated by caregiver control and pressuring override children’s internal hunger and satiety regulation and have been associated with feeding refusal, excessive snacking, higher intake of sugar-sweetened beverages, and overresponsiveness to food cues [40].

### Criterion 3: quality

A major challenge in the implementation of large-scale programs is ensuring quality, which is necessary for programs to achieve their intended goals without causing harm. Quality includes programs that are safe with a workforce that is trained, supervised, and supported. Programs with high quality are more effective in meeting their objectives than poor-quality programs [41,42], positively impact children and caregivers’ health, and, consequently, earn the trust of participants and communities [43]. Quality is assessed and maintained through monitoring and accountability.

Quality is a critical consideration in the transition from evidence-based interventions to large-scale programs. Although evidence-based interventions in nutrition and child development are widely available [[43], [44], [45]], many were implemented under controlled research conditions that focused primarily on the theory-of-change and research impact and were implemented by research assistants or highly supervised community health workers (CHWs). In contrast, controls are often loosened in the real-life conditions of large-scale programs; attention is shifted to criteria necessary to maintain, enhance, and sustain the program’s implementation, and the original impact may be diminished [18]. Successfully scaling interventions to large-scale programs requires attention to program quality, often measured through monitoring accountability [46]. An analysis by the Inter-American Development Bank highlighted the challenges in ensuring that quality is maintained in large-scale programs and called for a learning cycle that analyzes program successes and failures to understand the program and ecological criteria that contribute to long-term outcomes [47].

For example, a cluster-randomized trial was conducted to evaluate the impact of adding lipid-based nutrient supplementation and child development–oriented home visits to an existing, large-scale, community-based nutrition program in Madagascar among children aged 6 to 18 mo [48]. The authors attributed the lack of findings on development to limited take-up of behavior-change messages and delivery challenges facing CHWs, highlighting the importance of ensuring that messages and delivery strategies are culturally and developmentally appropriate and that CHWs receive supervision, support, and a manageable workload.

#### Training, supervision, and workforce support

Training programs to ensure that the entire workforce has competencies in their responsibilities and in the overall goals of the program are critical components of quality. For example, in nutrition and child development programs, all personnel should have basic competencies in child development, learning, and infant and young child feeding practices. Such resources include UNICEF’s Counselling Cards for Community Workers [49] and Zero-To-Three’s Defining Competencies for the Early Childhood Workforce [50]. In addition to initial training and periodic updates when new training methods are available, the workforce requires supportive supervision, opportunities for workforce growth and development, and a realistic workload [51]. The competence and enthusiasm of the frontline workforce in working with families and children are important aspects of quality and of the impact of programs on children’s nutrition and development.

#### Resources for delivery

The assurance that quality resources are available is critical because resources represent the materials that are necessary to implement programs and may include lesson plans, evaluation materials, food, play materials, and other logistical items. Large-scale programs often require supply chains and systems for procuring, monitoring, storing, delivering, and refreshing resources for programs, as well as ongoing funding to ensure their availability. For example, in addition to providing children with nutritious school meals, Home-Grown School Feeding programs in Ethiopia, Bolivia, Brazil, and Cambodia use local ingredients, farmers, and distributors, thereby providing quality ingredients and contributing to the local economy by sourcing resources [52].

#### Monitoring

Monitoring is a necessary aspect of quality because monitoring evaluates whether programs have been implemented as planned and whether decisions are based on data [53]. Monitoring enables program leaders and implementers to assess whether the program goals are being met and identify shortcomings. For example, if the number of anticipated families and communities have not been reached and are not enrolled or participating, corrective action, such as focused outreach, may be necessary. Monitoring is closely aligned with accountability and flexibility. Outcomes from monitoring are shared throughout the system to promote collaborative problem solving, learning, and harmonization across criteria and to facilitate necessary corrections or modifications to assure high-quality programs [53].

### Criterion 4: engagement

Ensuring community engagement in programs for young children is critical to establish mutual trust and collaboration, to identify and overcome barriers to program utilization, and to help connect families to services. A World Health Assembly resolution in 2022 [54] highlighted the central role of community stakeholders in clinical trials [55], which included key messages tailored to specific audiences and presentations with opportunities for questions and feedback.

The WHO recommends that large-scale nurturing care programs to improve children’s nutrition and development be implemented through government systems to align with government priorities [24]. The Aarambh program (Joyful Beginnings of Life), a primary initiative of the Department of Women and Child Development for Maharashtra, is an example of such a program. The program was launched by the Mahatma Gandhi Institute of Medical Sciences and UNICEF, initially in 2 districts of Maharashtra (Aurangabad and Yavatmal), and subsequently throughout the entire state of Maharashtra (population 129 million; 1.6 million children aged 0 to 3 y) [56]. Based on nurturing care and grounded in the socioecological model [57], the Aarambh program developed a model of services that uses data from multiple levels of the ecology to evaluate the program’s feasibility and impact. By including substudies and pilots, the program developers have implemented a learning model that enables them to engage primary and additional caregivers in planning and services, to ensure that the programs include age-appropriate activities for caregivers (including fathers) and families, and to implement flexibility in delivery platforms (including home visits, group sessions, and community activities) [58]. The program has found reductions in the proportion of children with underweight, stunting, and wasting, along with improvements in children’s development [58].

Engagement often includes government support, with advantages that include funding, sustainability, and collaboration across multiple sectors. However, government support may also include inadequate funding, excessive regulations, and upheaval, especially if policies change in response to changes in political control [59]. Thus, a balanced portfolio of engagement, including governments, along with other donors, communities, and families, is often recommended to ensure that program goals are aligned with current trends and demands [59].

### Criterion 5: rigorous and easy-to-use outcome assessments

Measurement and metrics are critical for the implementation and monitoring of programs and policies. Measurement of length/height of young children is often used as a summary indicator to assess growth and nutritional well-being. Practical field guides to assess child weight, length, and upper arm circumference at the population or community level are available [60]. Anthropometric measurements obtained in programs can be compared against global standards, established by WHO, to describe the proportion of children who are experiencing stunting, underweight, wasting, and overweight [61]. Nutritional deficiencies are assessed through physical examinations, dietary assessments, and biochemical tests. Assessment of anemia prevalence can be measured by a portable device that uses a single drop of blood to assess hemoglobin [62]. Global guidance on how to assess complementary feeding practices of children 6 to 23 mo-of-age is also available and widely used [63].

Child development can be measured through the WHO-endorsed Global Scales for Early Development (GSED), a newly developed open-access package that has been considered valid when compared with gold standard measures in multiple countries [64]. GSED provides a standard method to measure childhood development (ages 0–36 mo) globally [[65], [66], [67]] for population monitoring or program evaluation. The GSED Short Form is administered through caregiver-report and a Long Form is administered through direct observation. Both instruments are easy to administer through an app; include items that represent motor, cognitive, language, and socioemotional development; and produce a common metric, the developmental score (D-Score), which is an interval scale based on item difficulty that can be converted to an age-adjusted scale (DAZ) to compare children’s development over time and across contexts [68].

Families and caregivers may also be interested in aspects of children’s development that are not captured by the GSED, such as their curiosity, social development, or engagement in family activities. By clarifying caregivers’ goals through formative analyses, program planners can incorporate culture-relevant experiences into programs and measures of program impact on culture-relevant outcome measures.

In addition to focusing on program impact, addressing participants’ satisfaction, level of participation, and program suggestions are often helpful in future planning [69]. Ongoing review and designing evaluations for dissemination by sharing findings with stakeholders, including communities, are important activities for receiving timely feedback to improve project relevance, fostering trust, and enhancing the likelihood of continual program funding [70].

### Criterion 6: sustainability

Sustainability is an essential aspect of large-scale multicomponent programs for thriving because it illustrates the case for investing in children thriving. The return on investment for early childhood programs has been clearly demonstrated for nutrition and early child development programs [71,72]. Continuous funding is essential to avoid disruption of services, and long-term financial support should be planned from the onset as many projects fail once initial funding ends [73]. Multiple, rather than single, funding sources, including funding from collaborating communities, enhance the likelihood of continuous funding as multiple stakeholders are engaged and vested in program inputs and outcomes. The risks of a singular source of funding were driven home by the abrupt cessation of most projects funded by the United States Agency for International Development (USAID) in 2025. Many countries in LMICs relied on USAID grants to fund their community health systems, feeding programs, and HIV prevention and treatment programs, leading to catastrophic disruptions and devastating effects on mortality [74].

Continuous funding is facilitated by having quality programs and alignment between large-scale program goals and policies. Updating policies and implementation practices are primary responsibilities of program leaders. However, policies are not always implemented or monitored as planned, or the evidence-base may change, requiring policies to be updated [75]. A critical aspect of the success and sustainability of large-scale programs to improve young children’s nutrition and development is to maintain alignment with existing policies and to update policies based on evidence [76].

Laws can provide stability by bringing interested parties together to plan and establish programs for implementation [76]. For example, Ecuador has been engaged in a plan to promote early childhood development and education [77]. In 2025, Ecuador passed the Ley Orgánica para Prevenir y Erradicar la Malnutrición (Law for Preventing and Eradicating Malnutrition), in response to concerns about the prevalence of malnutrition. The law was followed by a National Intersectoral Strategic Plan to prevent malnutrition. In 2025, Ecuador also passed the Ley Orgánica de la Primera Infancia (Law on Early Childhood), in response to recommendations to broaden the perspective to include other aspects of children’s early development. The government is developing a National Early Childhood Plan to address child malnutrition and early child development comprehensively, in line with recommendations from the NCF [77]. Assuring alignment with comprehensive practices, policies, and laws on children’s early development and adhering to the criteria for large-scale multisector can facilitate funding, a critical aspect of sustainability.

### Management team

A management team, comprising representatives of the partnering sectors, program organizers and implementers, and participating communities, coordinates the system of criteria for implementation of large-scale programs to improve children’s nutrition and child development. The management team assumes overall responsibility for the operational and fiscal aspects of the program [78]. To fulfill that responsibility, management teams have 2 major roles: *1*) to ensure that the implementation of the large-scale program meets the established goals and is responsive to community needs by providing high-quality services and *2*) to coordinate data-driven accountability and communication that facilitates decision making related to program, policy, and law updates [78]. The management team sets operational and financial standards by ensuring that programs have operational manuals, monitoring and evaluation guidelines, and management strategies for program implementation, using available guides [79]. Community management team members are central as they can assess the community’s view toward the program, facilitate formal and informal collaboration within the communities, and stimulate community-led initiatives [79]. The management team addresses coordination through an interactive information system designed to share objectives regarding the 6 criteria, monitor routinely scheduled updates for each criterion, facilitate feedback, and plan for anticipated changes and updates. Through this process, the management team operates as a learning system (e.g., monitoring data, providing feedback, facilitating decisions, and recommending course changes as necessary). Systems changes may be necessary due to internal circumstances (e.g., program innovations, personnel changes, and updated electronic systems, etc.) and external circumstances (e.g., population shifts, natural disasters, and economic issues, etc.). By ensuring that the overall organizational system is dynamic, the management team facilitates internal and external communication, while ensuring financial sustainability, and updating programs, policies, and laws that are necessary to achieve the goal of improving young children’s nutrition and development.

## Discussion

Large-scale multicomponent nutrition and child development programs operate in communities, potentially in collaboration with other partners that provide support for young children and families, community centers, CHWs, parenting programs, and childcare centers. Community partners differ in their financial support, training, and focus but may be instrumental in collaborating with large-scale programs to support children’s nutrition and development. There are also potential threats to large-scale multicomponent programs, including community poverty and administrative barriers.

### Potential partners

Community centers, often supported by organizations such as Children’s International, refer to places for children to play and families to access food and services, such as health, education, and social services that support families with young children. Community centers support caregivers through demonstrations and opportunities where caregivers can gather in safe spaces with their children to share experiences and insights [80].

Many countries have CHWs supported by the health or child protection system, who provide home-based services, such as immunizations, nutrition, and treatment for infectious diseases. Trained CHWs earn community trust and are integral in supporting children’s health and nutrition [81]. CHWs may also have training in early childhood development and provide parenting support related to responsive caregiving and learning opportunities for children, as shown by the m2m (Mothers-To-Mothers) in eSwatini [82].

An analysis of studies of CHWs found strong endorsement for hiring CHWs from and by the community to increase their credibility and accountability to the communities [81]. CHWs are often viewed as liaisons with health systems by facilitating referrals and sharing information when appropriate. CHW retention is often driven by fair compensation and respect from families and from the system employing them, along with nonmonetary benefits, such as ongoing training and opportunities for advancement [81]. A qualitative study among CHWs in Rwanda found that CHWs valued the training, social status, and opportunities to help others and identified challenges such as aging equipment, discrepancies in financial reimbursements, poverty, and lack of formal workspaces or working hours. CHWs’ recommendations included compensation, transportation (e.g., bicycles or motor bikes), equipment (e.g., smart phones), infrastructure (e.g., waterproof bags), and career advancement opportunities [83].

Caregivers of young children often prefer CHWs who provide practical nutrition and parenting guidance on caring for their young children, as well as a trusting relationship and support for caregivers who may feel overburdened by daily caregiving tasks [83]. Caregivers look to CHWs to navigate relations with services and value CHWs who are from their community and understand their culture and the demands of daily life. Thus, effective CHWs would benefit from training in communication and social support for caregivers, along with training in children’s nutrition and early development.

Parenting programs in LMICs, primarily directed toward caregiving and children’s development, are often supported by nongovernmental organizations, governments, and research-based organizations. Parenting programs operate through multiple platforms, including home visits, group sessions in clinics and community sites, on-line individual and group sessions, and text messages. Mounting evidence suggests that caregivers appreciate parenting programs and children experience improvements in their cognitive, language, and motor development across multiple platforms, with programs that include both individual and group options having the greatest impacts [8,84,85]. Although there are outstanding implementation questions regarding the curricula, duration, and long-term impact, parenting programs can be scaled to reach caregivers and children at greatest need, where the programs are likely to be most effective [86].

In response to women’s increased role in the workforce in LMICs, there are urgent calls for childcare centers for young children [34]. Countries are increasingly initiating municipal childcare centers for low-income families. A recent review of childcare centers for children aged <3 y in LMICs found positive associations between childcare attendance and children’s growth, nutrition, and development [87]. Supporting healthy food in childcare centers is a potential opportunity to buffer the effects of food insecurity and to ensure that children experience healthy food options [88]. Investments in high-quality childcare programs that support women in the workforce and promote thriving by supporting children’s nutrition and child development may provide opportunities for large-scale programming.

However, even a program created with positive intentions to fill critical needs can lead to unintended consequences if there is insufficient attention to quality, as illustrated by a universal childcare program for young children in Quebec introduced in 1997. To enable mothers to enter the labor market, planning was limited, and the childcare program was implemented and expanded quickly at a very low cost [89]. Follow-up evaluations found that families and the community experienced economic benefits as women entered the labor force [90]. However, the duration of childcare program participation was negatively associated with school-age children’s and adolescents’ scores on self-reported health and life satisfaction [89], particularly among those from disadvantaged backgrounds [91]. These findings illustrate the need to consider programs’ economic impact and mothers’ workforce participation, along with children’s needs for high-quality programs.

### Potential threats

The potential threats to the implementation of large-scale programs to improve young children’s nutrition and development are often based in the ecology surrounding the program, ranging from families to communities and countries [92]. Threats can emanate from multiple levels, such that the lack of mutual trust between governing powers and programs can undermine programs, even for programs that are successful in supporting children’s nutrition and development. In contrast, trust and alignment between governing powers and programs can be mutually beneficial, enabling governments to point to reductions in nutritional deficiencies and increases in children reaching their developmental potential as indicators of successful government [69].

Fragile states, including countries or communities experiencing conflicts, natural disasters, and geopolitical and humanitarian crises may be unable to provide the structures, services, and safety net programs for children to thrive. In some cases, international agencies have provided assistance. For example, in conflict areas of Bangladesh, Syria, and Venezuela, the International Rescue Committee implemented Reach Up, an early childhood development program, emphasizing the importance of cultural adaptation including needs of children and caregivers, the integration of child and family safety with complementary services, and designing for eventual scaling to a large-scale program [93].

At the household level, millions of young children are raised in underresourced conditions of poverty that threaten thriving by impacting caregivers’ abilities to provide nutrition and opportunities for learning and responsive care that children need [94]. An analysis of 49 nationally representative surveys in LMICs showed that in most countries <50% of children 6 to 23 mo-of-age met the definition of minimum dietary diversity (consuming food from 5 of 8 food groups the preceding day), with stark disparities in minimum dietary diversity and dietary quality by wealth, urban and rural settings, and maternal literacy [95]. The consequences of early life poverty undermine children’s long-term health and human capital, based on national surveys and birth cohorts from LMICs [96]. Programs to alleviate community poverty through economic development or improved access to health and social services or childcare along with increasing maternal social capital may have downstream benefits on children’s nutrition and child development [97].

Household poverty often occurs along with other caregiver challenges, including limited education and vocational skills, risk of negative health conditions [98], and stress, financial strain, and depression [99]. These conditions can impact caregivers’ time and psychological availability to interact with their young children. When caregivers are unresponsive or unengaged, children do not experience the emotional feedback and exchanges needed to develop healthy cognitive and socioemotional development [37]. Strength-based approaches that focus on caregivers’ inherent strengths, resilience, and aspirations for their children, rather than their deficits and problems, can empower caregivers to strengthen their engagement with their families and children [100].

Conditional cash transfers (CCTs) linked to children’s health services may be an effective strategy in resources-limited communities. An integrated program in Tanzania that combined CCTs with home visiting by CHWs found significant improvements over control in children’s cognitive, language, and motor development and significant effects in linear growth in multivariable analyses [101]. The CCT was linked to attendance at routine antenatal care and well-child health and growth monitoring clinic visits. The findings, although modest, are encouraging because they illustrate the benefits of incorporating CCTs to promote healthcare visits with home visits to promote children’s nutrition and development. Additional research is needed to identify methods to facilitate healthcare visit attendance and promote children’s nutrition and child development that can be implemented into large-scale programming. Partnerships with economic support or social protection programs may be an innovative strategy to alleviate poverty and to promote children’s nutrition and development.

Administrative barriers to the implementation of large-scale multicomponent programs may include inadequate or intermittent funding, poor coordination across sectors, inadequately trained and supervised staff, and high workloads. In addition, program administrators and staff may have biases against participants based on race, ethnicity, nationality, or immigrant status that limit eligibility and enrollment [102]. Other barriers may include lack of resources and materials to conduct the programs, lack of transportation, and physical location or program timing that are not easily accessible to participants. Finally, programs may overwhelm caregivers with too much or disjointed information, as was suggested in a trial of complementary feeding and play in India [103]. These barriers highlight the importance of considering how the ecological context can influence the operation and success of programs [92].

Funding, continuity, and sustainability are major barriers to large-scale programs for children. Fiscal support may be facilitated by having a diversified funding portfolio that includes government funds, supplemented by public–private partnerships, corporate sponsors, philanthropic organizations, and ongoing fund raising [92]. Continuity and sustainability not only require attention to program quality, including staff training and support, and reaching children needing the services, but also ensure that families, local communities, donors, and political leaders are engaged and aware of the impact that the program is having on children’s nutrition and development [69]. Recommendations are to initiate and maintain programs from a wide ecological perspective to ensure that a program’s recognition as a source of pride and global good for the children, families, and community at large is established and sustained [92].

## Conclusion and call to action

This study focuses on increasing global opportunities for children to thrive. Using the NCF [24] as a conceptual guide, the article provides rationale, evidence, and systems-based criteria for implementation and sustainability of large-scale programs to improve young children’s nutrition and development.

The focus on children from birth through 24 mo stems from biological and developmental factors. With a nascent neural system, early childhood is the ideal time to ensure that children receive caregiving that emphasizes their changing nutritional and developmental requirements, consistent with the principles of nurturing care that will enable them to thrive.

By applying the principles of implementation science [18,75] as a management guide, the criteria for implementation and sustainability of large-scale programs to improve young child nutrition and development are integrated with overall coordination through a management team. The successful implementation of both nutrition and child development interventions illustrates that the expertise is in place for the implementation of multicomponent large-scale programs for young children in disadvantaged circumstances. An increase in children thriving during early childhood increases their likelihood of reaching their developmental potential and subsequently greater human capital. The long-term benefits of thriving enhance health and quality of life and extend to include economic productivity and sustainability for children, families, and countries.

## Author contributions

The authors’ responsibilities were as follows—MMB and CKL conceptualized the paper and participated equally in manuscript preparation and review. They both read and approved the final manuscript.

## Funding

The authors reported no funding received for this study.

## Declaration of generative AI and AI-assisted technologies in the writing process

During the preparation of this work, the authors used GEMINI to identify potential references. The authors reviewed all references and take full responsibility for the content of the publication.

## Conflicts of interest

MMB is an Editorial Board Member for the *Journal of Nutrition* and played no role in the Journal’s evaluation of the manuscript. The other author reports no conflicts of interest.
